# Testing Longitudinal Relationships between Internet Addiction and Well-Being in Hong Kong Adolescents: Cross-Lagged Analyses Based on three Waves of Data

**DOI:** 10.1007/s12187-017-9494-3

**Published:** 2017-09-16

**Authors:** Lu Yu, Daniel Tan Lei Shek

**Affiliations:** 1 Department of Applied Social Sciences, The Hong Kong Polytechnic University, Room HJ430, Hunghom, Hong Kong, People’s Republic of China; 2 Department of Applied Social Sciences, The Hong Kong Polytechnic University, Room HJ407, Hunghom, Hong Kong, People’s Republic of China; 3 Centre for Innovative Programmes for Adolescents and Families, The Hong Kong Polytechnic University, Hong Kong, People’s Republic of China; 4 Department of Social Work, East China Normal University, Shanghai, People’s Republic of China; 5 Kiang Wu Nursing College of Macau, Macau, People’s Republic of China; 6 Division of Adolescent Medicine, Department of Pediatrics, Kentucky Children’s Hospital, University of Kentucky College of Medicine, Lexington, KY USA

**Keywords:** Internet addiction, Quality of life, Hong Kong, Chinese adolescents, Longitudinal design

## Abstract

Using a panel design, this study examined the prospective relationships between Internet addiction and life satisfaction as well as hopelessness in a representative sample of Hong Kong adolescents. Starting from 2009/10 academic year, 3328 Secondary 1 students in 28 secondary schools in Hong Kong participated in this longitudinal study (Mean age = 12.59 years; SD = 0.74 years). All participants responded to a questionnaire that includes the Internet Addiction Test, Life Satisfaction Scale, and Hopelessness Scale on a yearly basis. Cross-lagged analyses based on three waves of data collected during three junior adolescent years showed that Internet addiction measured at Time 1 predicted poor life satisfaction and hopelessness at Time 2, but not vice versa. Similarly, Internet addiction at Time 2 predicted low life satisfaction at Time 3, and the cross-lagged effects of life satisfaction and hopelessness on Internet addiction from Time 2 to Time 3 remained non-significant. The findings support the thesis that poor personal well-being in adolescents is the consequence rather than the cause of Internet addictive behaviors. To improve quality of life and prevent suicidality in adolescents, strategies that help reduce addictive behaviors related to the Internet should be considered.

## Introduction

The world has entered an Internet era where connected electronic devices have been playing a gradually important role. From 1995 to 2016, the percentage of the world population who has access to the Internet has increased dramatically from less than 1% to approximately 46% (International Telecommunication Union 2016). While the use of the Internet has fundamentally changed the ways people live their lives, addictive behaviors related to Internet are fostered. In 1995, Goldberg (1995) used criteria that define substance dependence in the Diagnostic and Statistical Manual (4th edition) (American Psychiatric Association 1994) to describe problematic Internet use behavior including the following core symptoms: tolerance (need for longer periods of time online), withdrawal symptoms when reducing Internet use, lack of control on Internet use, continuation of Internet use irrespective of problem awareness, large amounts of time spent online, relapse, and negative consequences. In the same year, Young (1996) and Griffiths (1996) reported case studies on individuals who experienced such symptoms related to uncontrollable Internet use, which laid the foundation of empirical research into this area. A term, Internet addiction (IA), has been coined to refer to an individual’s incapability to control his/her Internet use, which ultimately leads to impairment in one’s daily life and psychological distress (Young 1998). Although other terms have also been adopted (e.g., pathological Internet use, Internet abuse, problematic Internet use, etc.) by different researchers, “Internet addiction” would be used in the present paper for consistency.

Based on these initial efforts, the phenomenon of Internet addiction has attracted intensive research attention during the last two decades and the number of empirical studies into this field has greatly increased (Dalal and Basu 2016). Research findings consistently showed that Internet addiction risk remains to grow particularly in adolescents across the world, although large variance of the reported occurrence rates was found (Shek et al. 2016; Young and Nabuco de Abreu 2010). Based on a systematic review of large-scale empirical studies published after 2000, researchers revealed that the occurrence rates of Internet addiction ranged from 0.8% to 26.7% in adolescents (Kuss et al. 2014). It is believed that the varied prevalence rates are mainly due to different Internet penetration rate in different areas, diverse measurement instruments, and miscellaneous cut-offs adopted to demarcate Internet addiction. Furthermore, many researchers and clinicians have found that Internet addiction symptoms are similar to other addictive disorders (such as instant gratification caused by online activities that change moods) and compulsive disorders (e.g., negative repercussions), and argued for including Internet addition in DSM-V as a distinct diagnosis. Although Internet addiction is not officially recognized as an independent disorder, one related condition entitled Internet Gaming Disorder has been included as a “condition for further study” in DSM-V (American Psychiatric Association 2013). Despite continuous controversies on this issue, there is a general recognition among helping professionals that no matter how Internet addiction is classified, people with Internet addiction needs need to be treated (Pies 2009).

With respect to Hong Kong, according to a report in 2004 (Tsuen Wan Centre 2004), around 18.8% to 35.8% of secondary school students, and 37.0% of university students were at high risk of Internet addiction. Based on a more stringent cut-off, Fu et al. (2010) reported that approximately 6.7% of Hong Kong adolescents (aged 15–19 years) showed five or more symptoms of Internet addiction. More recently, Shek and Yu (2016) found that the prevalence rates of Internet addiction ranged from 17% to 26.8% in Hong Kong high school students using Young’s IAT. It was also revealed that the occurrence rates of Internet addiction first increased and then gradually declined during adolescent years (Shek and Yu 2016).

While a consensual set of criterion for Internet addiction remains lacking and controversy exists in whether Internet addiction shall be considered a separate medical condition, empirical findings generally suggest that addictive behaviors related to the Internet has become an emergent problem among young people, which deserves more attention from researchers and professionals in the society (Chak and Leung 2004; Fu et al. 2010; Kuss et al. 2014; Shek and Yu 2012, 2013). Research has evidenced the pervasive negative impact of uncontrollable Internet use on young people’s physical health, academic achievement, family and other social relationship, and psychological well-being (Engelberg and Sjoberg 2004; Kim et al. 2006; Lin et al. 2013; Odaci and Çelik 2013). Besides, comorbidity between Internet addictive behaviors and other mental health problems has been reported (e.g., Byun et al. 2009; Ko et al. 2008; Shapira et al. 2000). Scholars have also warned that Internet addiction leads to loss of productivity in organizations without related regulation policies (Yellowlees and Marks 2007; Young and Nabuco de Abreu 2010). To prevent and tackle these problems effectively, there is an urgent need to further elucidate the mechanism that underlying the development of Internet addiction.

One of the most studied areas in Internet addiction research is the relationship between Internet addiction and personal well-being. Specifically, it has been widely found that Internet addiction is negatively related to life satisfaction, the cognitive component of subject well-being. According to Diener (1984), life satisfaction is defined as the overall assessment of one’s quality of life based on an individual’s personal and subjective judgment and criteria, which reflect the extent to which an individual is satisfied with his/her life as a whole. Based on a meta-analysis of studies conducted in 31 nations of seven world region, Cheng and Li (2014) found that “Internet addiction prevalence is inversely associated with the quality of life as reflected by both subjective (life satisfaction) and objective (quality of environmental conditions) indicators” (p. 755). Similar results were published by researchers in different professional disciplines (Cao and Su 2007; Ko et al. 2008; Fu et al. 2010). However, the directions of the causal relationships amongst Internet addiction and life satisfaction remain unclear. Hence, there is an urgent need to clarify this theoretical issue.

Another indicator of subjective well-being is hopelessness which refers to negative views or expectancies with respect to the future (Beck et al. 1985). People with high level of hopelessness generally believe that good things will never happen to their lives and they can do nothing to change the situation. According to the learned hopelessness theory, perceived negative life event together with an individual’s maladaptive inferential style contribute to the development of hopelessness. The maladaptive inferential style includes a) attribution of negative events to stable, global, and internal causes; b) belief that negative life events lead to aversive consequences, and 3) drawing negative inferences about the self (Abramson et al. 1989). A recent review (Lester 2013) revealed that from 1978 to 2010 there was an increase in hopelessness among American undergraduates over the years, indicating that nowadays young people may become more depressed and hopeless which deserve further investigation.

Studies on the relationship between hopelessness and Internet addiction are sparse, although many researchers have found that Internet addiction was significantly associated with symptoms of depression. For example, Caplan (2003) reported that depression and loneliness predicted Internet use problem. Based on a cross-sectional study, Alpaslan et al. (2016) reported that hopelessness was higher among major depressive disorder patients with Internet addiction than patients without Internet addiction. In another study (Velezmoro et al. 2010), perceived hopelessness was found predictive of Internet abuse for non-sexual instead of sexual purpose. These studies generally showed that Internet addicts tend to have a higher level of hopelessness than individuals without Internet addiction.

According to the cognitive-behavioral theory (Davis 2001) and the problematic psychosocial predisposition model (Caplan 2003), psychosocial maladjustments lead to maladaptive cognitions such as the belief that one can solve his/her problem via Internet surfing. Internet addiction, therefore, represents an adaptive “self-soothing” which satisfies one’s unmet psychosocial needs and helps one to avoid/alter discomfort feelings related to the underlying psychological problems. Although excessive Internet use may further worsen one’s problems and create new problems, it is believed that people who are addicted to the Internet would have a certain degree of pre-existing psychological inadequacy. Therefore, Internet addiction should be considered a secondary manifestation (i.e., effects) of previously existing low level of personal well-being (e.g., low life satisfaction or high sense of hopelessness) instead of the cause of one’s problems (Caplan et al. 2009; Chak and Leung 2004; Lo et al. 2005).

On the other hand, some researchers argued that problematic Internet use causes the deterioration of one’s social and emotional competence, which in turn impair one’s well-being (Beard 2005; Morahan-Martin and Schumacher 2003; Young and Rogers 1998). According to the displacement theory, the Internet could undermine one’s social development through occupying necessary time to spend with family and friends (Kraut et al. 1998). Decreased social interaction in real world caused by excessive Internet use may result in social isolation, depression, and loneliness. It was reported that some heavy Internet users indulged in online relationship or extramarital affairs which led to family problems and difficulties in real-life social relationships (Young 1998). There were also findings showing that Internet use behavior such as gaming negatively affected marital satisfaction (Ahlstrom et al. 2012).

While a number of studies have been conducted to address the relationships between Internet addiction and personal well-being, the majority was cross-sectional and available findings based on a limited number of longitudinal studies are inconsistent. It was found that pre-existing psychosocial problems, such as suicidal ideation and loneliness predicted Internet addictive behaviors later (Gentile et al. 2011; Koronczai et al. 2013; Yao and Zhong 2014). In contrast, some researchers reported that the time spent online was negatively related to one’s quality of life (Moody 2001). People with Internet addiction problem reported lower happiness and life satisfaction (Kraut et al. 1998; Kowert et al. 2015). There are also findings supporting a reciprocal relationship between Internet addiction and psychological well-being (Senol-Durak and Durak 2011). As such, the existing studies cannot provide sound evidence as to whether Internet addiction causes poor personal well-being or vice versa.

Taking previous research findings into account, the present study aimed to examine the causal relationship between Internet addiction and two personal well-being indicators (life satisfaction and hopelessness) among a representative sample of Hong Kong adolescents over a three-year period. Several cross-lagged models that hypothesize different relationships between Internet addiction and personal well-being indicators over time would be examined after controlling for the possible effects of demographic factors (Kuss et al. 2014). In cross-lagged panel models, both autoregressive effects that describe the stability of Internet addiction and personal well-being constructs across different time points, and the cross-lagged effects that hypothesize the effects of one construct on another from one occasion to the next can be examined simultaneously. This helps minimize the bias in estimating the hypothesized cross-lagged effects.

The present study examines four competing hypotheses regarding the direction of effects between Internet addiction and personal well-being: 1) Internet addiction and personal well-being do not influence each other directly, but share variance caused by unmeasured factors (i.e., stability model); 2) Personal well-being indicators have direct and longitudinal effects on Internet addiction; 3) Internet addiction has a direct effect on personal well-being, or 4) Internet addiction and personal well-being indicators demonstrate reciprocal and longitudinal effects. Findings of this pioneer study are expected to deepen our understanding about the causes and effects of Internet addiction in adolescents. The results would also shed light on the development of theoretical models on Internet addiction as well as prevention and intervention programs to promote young people’s personal well-being.

## Methods

### Participants and Procedure

The present study was part of a large survey project that traces the development of secondary school students in Hong Kong. Based on a list of all secondary schools in different districts of Hong Kong provided by the local Educational Bureau, 28 secondary schools were randomly selected to join the project, including 5 schools from Hong Kong Island, 7 schools from Kowloon, and 16 schools from New Territories. According to Shek et al. (2014), the demographic attributes of the present sample compared favourably with those in the general high school student population in Hong Kong. Starting from 2009/10 academic year, we invited all students studying in Secondary 1 from the 28 secondary schools to participate in the study. During their secondary school lives, the participants were surveyed on a yearly basis in terms of multiple aspects of their development including Internet addictive behaviors, life satisfaction, hopelessness, family processes, and multiple indicators of positive youth development qualities. Before each survey, consents from schools, parents, and respondents were obtained. The participating students were reassured of the confidentiality of their personal information. At least one research staff administered the survey in classroom settings and answered possible questions raised by the participants.

The present study was based on three waves of data collected during the participants’ junior secondary years, i.e., Time 1: when students just entered into secondary school (Secondary 1; *n* = 3328); Time 2: when students had spent one year in secondary school (Secondary 2; *n* = 3638); and, Time 3: when students would be graduated from junior secondary school (Secondary 3; *n* = 4106). Across three waves, 2023 students were successfully matched with complete data, which included 1040 male students, 959 female students, and 24 students who did not indicate their gender. Basic demographic characteristics of participants were summarized in Table 1. Statistical analyses that compared participants who only completed the first survey and those who completed the questionnaire at all waves (i.e., included in the present study) showed no significant difference in gender ratio and family economic status. Participants included in the present study (age = 12.53 ± 0.66 years) were slightly younger than participants who only completed the survey at Time 1 (age = 12.59 ± 0.74 years), *t* = 2.99, *p* = .01. In terms of the variables under focus of the present study, no significant differences were identified in life satisfaction (*t* = −1.34, *p* > .05) and hopelessness (*t* = −.63, *p* > .05), while participants who completed questionnaires at all waves reported higher scores on Internet addiction than participants only completed Wave 1 survey (*t* = −3.89, *p* < .001).

**Table 1:** Demographic profile and descriptive statistics of the key variables across two waves

	Group 1	Group 2	Group 3	Group 4	Comparison between Group 1 and Group 4
	Wave 1 (N ^a^ = 3328)	Wave 2 (N ^a^ = 3638)	Wave 3 (N ^a^ = 4106)	Matched cases (*N* = 2023) ^b^	
Age	12.59 ± 0.74	17.33 ± 0.72	14.65 ± 0.80	12.53 ± 0.66	*t* = 2.99, *p* = .01
Gender					*x* ^*2*^ = 0.02, *p* = .88
Male	1719 (52.2%)	1864 (52.1%)	2185(53.7%)	1040 (52.0%)	
Female	1572 (47.8%)	1716 (47.9%)	1885(46.3%)	959 (48.0%)	
FES					*x* ^*2*^ = 0.62, *p* = .43
CSSA	225 (6.8%)	208 (5.8%)	212(5.2%)	129 (6.4%)	
Non-CSSA	2606 (79.1%)	2932 (81.2%)	3308(81.4%)	1636 (80.9%)	
Unknown	465 (14.1%)	472 (13.1%)	545(13.4%)	258 (12.8%)	

*FES* Family Economic Status

^a^The numbers were based on the participants who completed the survey at different waves

^b^Scores in this column were measured at Wave 1 from this group of participants

### Instruments

#### Internet Addiction

Adolescents’ Internet addictive behaviors were measured by Young’s 10-item Internet Addiction Test (IAT) has been translated into Chinese and validated in multiple samples of Hong Kong adolescents (e.g., Shek et al. 2008; Shek and Yu 2016). Respondents were asked to answer whether they had displayed the described behaviors related in the past year. The number of addictive behaviors related to the Internet reported by participants was used in the present study as an indicator of Internet addiction. Previous studies have provided evidence for the good psychometric properties of the IAT (Shek and Yu 2012). For the present study, Cronbach’s alpha of the IAT at the three time points ranged from 0.77 to 0.81 and the mean inter-item correlation coefficients were all above .26 (see Table 2), suggesting good internal consistency of the scale (Clark and Watson 1995).

**Table 2:** Cronbach’s alpha coefficients of scales at each time point of the three waves (n = 2023)

Scale	Wave	Cronbach’s alpha	Mean inter-item correlation
IAT	Time 1 (Wave 1)	.77	.26
	Time 2 (Wave 2)	.79	.27
	Time 3 (Wave 3)	.78	.27
SWLS	Time 1 (Wave 1)	.85	.54
	Time 2 (Wave 2)	.87	.59
	Time 3 (Wave 3)	.87	.58
HOPEL	Time 1 (Wave 1)	.85	.54
	Time 2 (Wave 2)	.86	.56
	Time 3 (Wave 3)	.87	.59

#### Satisfaction with Life Scale (SWLS)

Students’ life satisfaction was measured by the widely used 5-item SWLS (Diener et al. 1985). Shek (1992) translated the questionnaire into Chinese to assess Hong Kong people’s global judgment on their quality of life. Participants were asked to rate themselves in terms of the five items on a 6-point Likert scale (1 = strongly disagree; 6 = strongly agree). The mean scale score of the SWLS (ranged from 1 to 6) was used in this study. At each time point, the SWLS showed good psychometric properties with Cronbach’s α ranged from .85 to .89 and mean inter-item correlation coefficient ranged from .54 to .62 (Table 2).

#### Chinese Hopelessness Scale (HOPEL)

The 5-item Chinese Hopelessness Scale (Shek 1997) modified from Beck et al.’s (1974) original scale was used to measure participants’ sense of hopelessness. Individuals were asked to evaluate the degree to which they would agree with each statement about their lives on a 6-point Likert scale (1 = strongly disagree; 6 = strongly agree). A sample item reads as “the future seems vague and uncertain to me”. In this study, mean score of the scale was used to indicate participants’ sense of hopelessness about their lives. Cronbach’s α was .85, .87, and .89 at the three assessment occasions, respectively.

#### Family Economic Status (FES)

Family economic status of the participants was assessed based on the self-reported information about whether the participant’s family receives Comprehensive Social Security Assistance (CSSA) or not. In Hong Kong, families receiving CSSA are generally considered having financial difficulties (Shek and Lin 2014; Shek and Tsui 2012). At the first wave of data collection, 79.1% of the students reported that their families did not receive CSSA, 14.1% of the students indicated unknown, and 6.8% reported receiving CSSA (Table 1).

### Data Analytic Plan

**Fig. 1:**
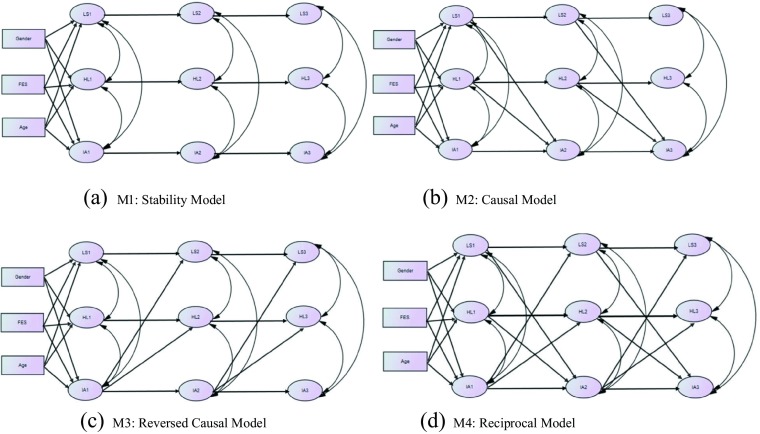
Hypothesized structural models

Structural equation modelling (SEM) with AMOS 23.0 software package was employed to examine the cross-lagged longitudinal model. First, the measurement models of the three latent variables, Internet addiction, life satisfaction, and hopelessness were tested at each wave. Second, four competing hypothesized structural models were tested using data collected at three time points when students were in Secondary 1, Secondary 2, and Secondary 3 to examine the proposed cross-lagged effects. The first model (M1) can be regarded as a stability model, which contains only the autoregressive effects of each latent variable across two waves, but did not contain any cross-lagged effects. The second model (M2) is a causal model which includes both the autoregressive effects as specified in M1 and cross-lagged effects from life satisfaction and hopelessness at an earlier time point (Time 1 and Time 2) to Internet addiction later (Time 2 and Time 3). The third model (M3) represents a reversed causal model including both the autoregressive effects and the cross-lagged effects from Internet addiction at earlier time points to life satisfaction and hopelessness later, i.e., reversed effects of the causal paths specified in M2. The fourth model (M4) is named as the reciprocal model combining M2 and M3, which assumes that there are reciprocal relationships between Internet addiction and the two personal well-being indicators over time. For each model, we allowed synchronous correlations among latent variables and covariation of the error terms of each indicator at Time 1 with the corresponding indicator at Time 2 and Time 3, as a common practice in longitudinal structural equation modeling (Gollob and Reichardt 1991). The four hypothesized models are illustrated in Fig. 1 (a-d).

Third, to avoid potential influence of demographic factors on the relationship between Internet addiction and personal well-being, participants’ gender (male = 1; female = 0), age, and family economic status (CSSA = 1; non-CSSA = 0) at Time 1 were included in the panel model as possible confounders as suggested by previous studies (Kuss et al. 2014; Yu and Shek 2013). It was assumed that these demographic factors were directly related to Wave 1 variables, and only indirectly linked to variables measured later through the test-retest correlation across waves.

## Results

Descriptive statistics of all variables under study were calculated and summarized in Tables 1 and 2. The pattern of cross-sectional and longitudinal correlations among variables was as expected based on the existing literature, with Internet addiction negatively associated with life satisfaction, and positively related to hopelessness both synchronously and longitudinally. Life satisfaction and hopelessness were negatively correlated.

Table 3 summarizes the goodness of fit indices of both measurement models and the four hypothesized structural models. It can be seen that all measurement models (MM1 to MM9) displayed a good fit to the data, suggesting that the assessment tools of life satisfaction, hopelessness and Internet addiction were valid and reliable across three waves (Anderson and Gerbing 1988). Results of goodness-of-fit indices of the four hypothesized structural models showed that the models fit the present three-wave data satisfactorily (*CFI* ≥ .95, *NFI* ≥ .92, *TLI* = .95, and *RMSEA* = .03). As all structural models are nested models, they were compared by chi-square difference tests (Bentler and Bonett 1980), and the results are presented in Table 3.

**Table 3:** Descriptive statistics of variables for participants who completed all six waves of questionnaire survey

Variables	Range	Mean ± SD	Skewness	Kurtosis	IA1	LS1	HL1	IA2	LS2	HL2	IA3	LS3	HL3
IA1	0–10	2.15 ± 2.25	1.19	0.92	–								
LS1	1–6	3.98 ± 1.05	−0.48	−0.05	−.31^**^	–							
HL1	1–6	2.59 ± 1.11	0.68	0.13	.26^**^	−.32^**^	–						
IA2	0–10	2.28 ± 2.33	1.16	0.82	.55^**^	−.16^**^	.21^**^	–					
LS2	1–6	3.85 ± 1.06	−0.46	−0.07	−.25^**^	.56^**^	−.30^**^	−.23^**^	–				
HL2	1–6	2.66 ± 1.10	0.56	0.04	.27^**^	−.31^**^	.47^**^	.29^**^	−.41^**^	–			
IA3	0–10	1.17 ± 2.17	1.66	1.55	.44^**^	−.13^**^	.14^**^	.56^**^	−.16^**^	.10^**^	–		
LS3	1–6	3.59 ± 1.05	−0.29	−0.37	−.22^**^	.51^**^	−.26^**^	−.16^**^	.61^**^	−.32^**^	−.18^**^	–	
HL3	1–6	2.67 ± 1.06	0.50	−0.01	.21^**^	−.29^**^	.43^**^	.26^**^	−.36^**^	.57^**^	.29^**^	−.39^**^	–

*IA1* Internet addiction at Time 1 (Wave 1); *LS1* Life satisfaction at Time 1 (Wave 1); *HL1* Hopelessness at Time 1 (Wave 1); *IA2* Internet addiction at Time 2 (Wave 2); *LS2* Life satisfaction at Time 2 (Wave 2); *HL2* Hopelessness at Time 2 (Wave 2); *IA3* Internet addiction at Time 3 (Wave 3); *LS3* Life satisfaction at Time 3 (Wave 3); *HL3* Hopelessness at Time 3 (Wave 3)

Scores of IA were based on the number of “Yes” answers from the *IAT* scale, i.e., the number of Internet addictive behaviors measured by *IAT*; scores of life satisfaction and hopelessness were calculated based on the averaged item scores of *SWLS* and *HOPEL*

**p < .001

First, the stability model (M1) without cross-lagged paths was compared to the causal model (M2) that specifies the cross-lagged effects of life satisfaction and hopelessness at Time 1 and Time 2 on Internet addiction at Time 2 and Time 3, respectively. The results showed no significant improvement (*Δx* ^*2*^ = 8.91, *Δdf* = 4, *p* > .05). Second, the reversed causal model (M3) with the cross-lagged effects of Internet addiction at an earlier time point (Time 1 and Time 2) on later life satisfaction and hopelessness (Time 2 and Time 3) provided a better fit to the data than the stability model (*Δx* ^*2*^ = 93.74, *Δdf* = 4, *p* < .001). Third, while the reciprocal model (M4) fit the data better than M1 (stability model) and M2 (causal model), this model did not significantly improve the model fit as compared with M3, the reversed causal model (*Δx* ^*2*^ = 8.57, *Δdf* = 4, *p* > .05). Therefore, M3 appeared to be the best fitting model in terms of parsimony, although M4 showed marginally significant improvement as compared to M3 (*p* = .04 using one-tailed test) which may also deserve attention. In other words, the data supported the hypothesis that Internet addiction causes low life satisfaction and high level of hopelessness in the future, but not vice versa (Table 4).

**Table 4:** Model fit indexes of measurement models and structural models (N = 2023)

Model	Description	*x* ^*2*^	*df*	*CFI*	*NFI*	*TLI*	*RMSEA*	Model comparisons	*Δx* ^*2*^	*Δdf*	*p*
MM1	IA Time 1	144.09	33	.97	.96	.96	.04	–	–	–	–
MM2	LS Time 1	6.2	4	1.00	1.00	1.00	.02	–	–	–	–
MM3	HL Time 1	1.4	3	1.00	1.00	1.00	.00	–	–	–	–
MM4	IA Time 2	154.59	33	.97	.96	.96	.04				
MM5	LS Time 2	18.2	4	1.00	1.00	.99	.04				
MM6	HL Time 2	4.7	3	1.00	1.00	1.00	.02				
MM7	IA Time 3	179.72	33	.97	.96	.95	.05	–	–	–	–
MM8	LS Time 3	7.6	4	1.00	1.00	1.00	.02	–	–	–	–
MM9	HL Time 3	11.5	3	1.00	1.00	.99	.04	–	–	–	–
M1	Stability model	4304.64	1794	.95	.92	.95	.03	–	–	–	–
M2	Causal model	4295.73	1790	.95	.92	.95	.03	M1 vs. M2	8.91	4	.06
M3	Reversed causal model	4210.90	1790	.96	.93	.95	.03	M1 vs. M3	93.74	4	.00
M4	Reciprocal model	4202.33	1786	.96	.93	.95	.03	M1 vs. M4	102.31	4	.00
								M2 vs. M4	93.40	4	.00
								M3 vs. M4	8.57	4	.07

*MM* Measurement model (e.g., *MM1* Measurement model 1)

**Fig. 2:**
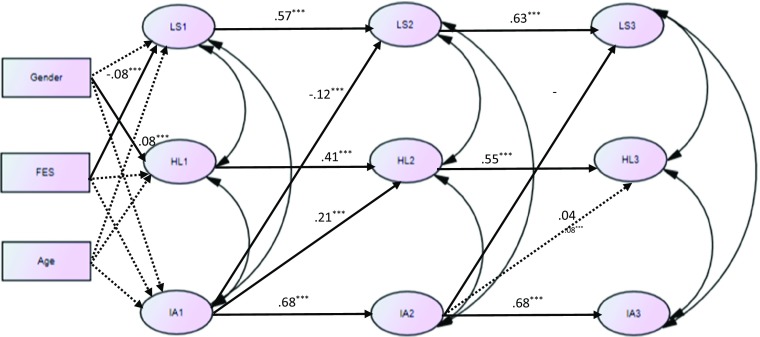
Reversed Causal Model (M3): Cross-lagged relationships between Internet addiction, life satisfaction, and hopelessness across three waves (N = 2023)

Figure 2 further showed the path coefficients of the supported reversed causal model (M3). First, at Time 1, gender (being male) was positively related to hopelessness (*β* = .08, *p* < .001) and low family economic status (receiving CSSA) was negatively related to adolescents’ life satisfaction (*β* = −.08, *p* < .001). Second, adolescents’ Internet addiction at Time 1 had a positive longitudinal cross-lagged effect on hopelessness at Time 2 (*β* = .21, *p* < .001), and a negative cross-lagged effect on their life satisfaction at Time 2 (*β* = −.12, *p* < .001), after controlling for their autoregressive effects and the influence by demographic variables. Third, from Time 2 to Time 3, Internet addiction negatively predicted life satisfaction (*β* = −.10, *p* < .01), while the prediction of hopelessness was not significant (*β* = .04, *p* > .05).

## Discussion

Most of the previous studies on the relationship between Internet addiction and personal well-being in young people have been based on cross-sectional design. As such, longitudinal data from a representative sample are necessary for researchers to understand whether poor well-being is a risk factor for youth Internet addiction or its consequence. The present study serves this purpose by examining the longitudinal relationships between Internet addiction and two personal well-being indicators, life satisfaction and hopelessness, in a large sample of Hong Kong adolescents.

Based on a three-wave cross-lagged panel design, the results supported a reversed causal model such that Internet addiction caused decreased personal well-being after the baseline status and the effects of gender, age, and family economic status were controlled for. The reciprocal model that hypothesized mutual influences was not supported. These findings provide new insights into the direction of relationships between Internet addictive behaviors and youth personal well-being. In contrast to cross-sectional studies, the use of panel design and structural equation modeling is a more rigorous approach to examine the issues of causality and reciprocity.

It was found that Internet addiction predicted adolescents’ low life satisfaction and high hopelessness longitudinally, but the cross-lagged effects of the two well-being indicators on Internet addictive behaviors were non-significant. While this finding confirms the negative relationship between Internet addiction and personal well-being, the direction of this association is only partially consistent with previously reported findings (Cao et al. 2011; Ko et al. 2008; Whang et al. 2003). For example, there were findings showing that adolescents with pre-existing psychosocial vulnerabilities are particularly susceptible to addictive involvement with Internet use (e.g., Lemmens et al. 2011a). A study by Bozoglan et al. (2013) revealed that low life satisfaction, low self-esteem, and high loneliness predicted Internet addiction in university students. In another longitudinal study (Lemmens et al. 2011b), lower psychosocial well-being was found to be a cause than a consequence of pathological computer uses and video gaming. Sun and Shek (2013) also reported that life satisfaction mediated the relationship between positive attributes and a list of youth problem behaviors in that positive judgment about life mitigates future problem behavior by enabling prospective positive youth development. These findings converged to suggest a causal pathway from lowered well-being to Internet addiction.

Meanwhile, quite a few scholars tend to believe that there is a reciprocal relationship between psychological well-being and Internet addiction: while one with poor well-being may intensively use Internet as a coping strategy to escape from the stress he/she experiences in reality, immersing oneself into the virtual world of the Internet actually creates more real life problems and feelings of loneliness which in turn worsen the individual’s personal well-being. Unfortunately, the reciprocal model did not gain much empirical support in this study.

There are several plausible explanations for the present findings. First, the findings can be regarded as evidence for the displacement theory. That is, young people who are addicted to the Internet put the first priority on their Internet use over other things and feel a sense of displacement when online. No matter whether the adolescent has pre-existing psychosocial condition or not, it is the displacement that isolates the individual from his/her real life that causes adjustment problems (e.g., family, study, physical problems) and a decreased level of well-being. For example, sleeping problems have often been reported as a result of Internet addiction (Chen and Gau 2016; Do et al. 2013), and lack of sleep is associated with a lower level of satisfaction with life (Piper 2016; Van Praag and Ferrer-i-Carbonell 2007) and a higher sense of hopelessness (McCall and Black 2013). As such, the physical problems caused by one’s overuse of the Internet could directly affect one’s quality of life.

Second, although pre-existing psychosocial problems such as depression, stress, and social anxiety may predispose adolescents to Internet addiction, the problems themselves may not be strong enough for adolescents to become addicted to the Internet. There are obviously other factors that contribute to the development and maintenance of Internet addiction. For example, high impulsivity of the individual (Lee et al. 2012), free access to the Internet (Young 2004), positive reinforcement of online behaviors (e.g., sense of achievement, decreased loneliness), a cognitive belief that Internet is a friend who soothes one’s distress (Davis 2001), etc. Without these factors, poor psychological well-being alone may not result in Internet addictive behaviors in adolescents. Third, it is also possible that the causal relationship between well-being and Internet addiction is moderated by other factors such as parental behavioral control. Researchers found that adolescents reported more monitoring behaviors by their parents tend to display less Internet addictive behaviors than did those with reporting less parental monitor (Li et al. 2013). Apparently, more in-depth research is needed to examine the potential effects of different moderators and to further test the reciprocal model which was found as marginally significant in this study. Furthermore, although the support for the reciprocal model is not strong, the marginally significant chi square difference suggests that there is a need to explore this model using more waves of longitudinal data.

The present findings have both theoretical and practical implications for researchers and practitioners working with youth. Theoretically, as very few studies have examined the longitudinal association between Internet addiction and hopelessness, the finding that Internet addiction increases adolescents’ feeling of hopelessness over time adds to the literature of this field. In particular, it suggests that personal well-being is not a significant factor leading to Internet addiction. One possibility is that those with high personal well-being may also be prone to Internet addiction. On the other hand, those who have low personal well-being may not have the energy to get addicted and they simply lack motivation to have prolonged engagement in the Internet. The present findings suggest that there is a need to look at the possible theoretical relationships between personal well-being and addiction.

Practically, the findings provide a new angle on how to promote adolescents’ personal well-being. In particular, researchers have argued that hopelessness is a significant predictor of depression and suicidality (Minkoff et al. 1973), and that hopelessness would lead to a series of hopeless deficits including passivity and lowered perseverance, anxiety and sadness, lowered self-esteem and inability to perceive the controllability of negative events. To reduce adolescents’ hopelessness and promote their personal well-being, strategies and tools that can help screen and treat Internet addictive behaviors should be considered. For example, studies have suggested that cognitive behavioural approach that specifically target Internet addiction can be useful in reducing the symptoms (King et al. 2011; Jorgenson et al. 2016; Winkler et al. 2013; Young 2007). Based on this approach, helping professionals in school or community settings can focus on monitoring adolescents’ Internet use behaviors (such as helping the adolescent record their own daily online activities), correcting adolescents’ distorted cognition about Internet, and teaching time management as well as goal setting skills. Multilevel intervention that incorporates both individual counselling and family intervention has also been found effective in reducing one’s time spent online and related psychosocial problems (Shek et al. 2009). When adolescents are less addicted to the Internet, they may be more likely to engage in real social interaction and build up social connection which can help promote a sense of hopefulness for the future in adolescents (Stoddard et al. 2011). Obviously, as other factors may contribute to Internet addiction (e.g., family processes), we have to examine these factors as well. Finally, different stakeholder including teachers, parents and the students themselves should be sensitive to the harmful consequences of Internet addiction. The present findings can be used to develop evidence-based prevention programs for Internet addiction.

Several limitations of the present study should be noted. First, although we have used cross-lagged modelling with longitudinal data collected over three years to infer causal relationships between Internet addiction and personal well-being in young adolescents, evidence based on studies with experimental design is needed to confirm such cause-and-effect relationship. Future studies may adopt randomized controlled trial to further test whether changing adolescents’ Internet addictive behaviors would increase their life satisfaction and decrease hopelessness. Second, while we controlled the effects of demographic factors by including the variables measured at Time 1 in the cross-lagged models, it was assumed that these factors would have direct influence on Internet addiction, life satisfaction, and hopelessness measured at Time 1, and only indirect effects on these constructs measured at later waves through their autoregressive effects. However, it is possible that demographic variables may change across time (e.g., family economic status) and there could also be synchronic association between demographic factors and these constructs at later time points. Therefore, future studies may include these factors as covariates at each wave when examining the relationship between Internet addiction and personal well-being. Besides, there were findings showing that the negative association between problematic Internet use and well-being was stronger in female adults than in males. It would be interesting to investigate possible gender difference in such relationship in adolescents by using multi-group structural equation modeling approach.

The final limitation is that the cross-lagged effects found in the present study were relatively weak, especially the effect of Internet addiction at Time 2 on hopelessness and life satisfaction at Time 3. One explanation may be that adolescents’ personal well-being in the last year of junior secondary school study is affected substantially by other factors than Internet addiction, such as stress of the entrance examination to senior secondary school. Therefore, the additional variance that can be explained by Internet addiction is limited. Also, the effects of Internet addiction on hopelessness and life satisfaction may be moderated by other factors, such as the students’ academic performance, which were not investigated in the present study. In future research, possible moderating effects of students’ academic results and perceived stress on the relationship between Internet addiction and personal well-being should be further examined. Although the effects found in the present study were moderate, the findings can be regarded as meaningful.
